# The Spread of Plasmidic AmpC in a General Lebanese Hospital Over Nine Consecutive Years and the Relationship With Restricted Isolation Protocols

**DOI:** 10.3389/fmed.2021.633783

**Published:** 2021-10-26

**Authors:** Mohamad Fleifel, Ahmad Machmouchi, Omar Alameddine, Kim Hoyek, Dimitri Melki, Elsa Hallab, Khalil Masri, Hiam R. Sidaoui, David Stockman, Ziad Daoud

**Affiliations:** ^1^Department of Internal Medicine, The Lebanese American University Gilbert and Rose-Marie Chagoury School of Medicine, Beirut, Lebanon; ^2^Faculty of Medicine and Medical Sciences, University of Balamand, Beirut, Lebanon; ^3^Centre Hospitalier du Nord Hospital, Department of Infectious Diseases, Zgharta, Lebanon; ^4^Clinical Microbiology and Infection Prevention, Michigan Health Clinics, Saginaw, MI, United States; ^5^Clinical Microbiology and Infection Prevention, Michigan Health Clinics and College of Medicine-Central Michigan University, Saginaw, MI, United States

**Keywords:** AmpC, ESBL, extended-spectrum β-lactamase, Lebanon, infectious disease, isolation protocol, plasmid, Enterobacteriaceae

## Abstract

**Background:** The dreaded bacterial infection by extended-spectrum β-lactamases (ESBL)-producers has always troubled the medical field whether on the public, scientific, or clinical levels. One of the lesser known β-lactamases, which is capable of hydrolyzing broad and extended-spectrum cephalosporins—i.e., cephamycins plus oxyimino-β-lactams—are the AmpC β-lactamases. This group, which has also been termed occasionally—and incorrectly—as ESBL Class C, confers resistance to β-lactamase inhibitors. The prevalence of plasmidic AmpC (pAmpC) strains is possibly still a matter of debate considering the unevenly matched data between phenotypically-detected and molecularly-detected pAmpC.

**Aim:** In the absence of any study in Lebanon addressing the AmpC, our intention was to determine the numbers and percentages of AmpC Enterobacteriaceae isolates, notably plasmid-mediated ones, across different wards at the Centre Hospitalier du Nord (CHN), Lebanon, and highlight the importance of infection control protocols.

**Materials and Methods:** Carriage and infection with pAmpC Enterobacteriaceae were retrospectively investigated between 2011 and 2015 and prospectively between 2016 and 2019 at the Centre Hospitalier du Nord Hospital, North Lebanon. The rise or decline in the numbers of such strains, in concordance with the allegedly intensive isolation of the patients, were analyzed.

**Results:** Intensive care unit (ICU) data shows an initial rise in infection isolates from 2012 to 2014 and in the carriage isolates from 2012 to 2013 with later notable overall decrease in the both isolates' numbers with the application of the isolation protocols at CHN from 2014 onwards. Floors 2, 3, and 4 seemed to house the bulk of the isolates as well.

**Conclusion:** Preventive measures, such as on-going surveillance of the hospital wards by specialized healthcare personnel and strict implementation of infection control practices, should be a top priority in any medical center in order to isolate such strains and try to put a limit for the development and the dissemination of any possible multidrug resistant strains.

## Introduction

Extended-spectrum β-lactamases (ESBL)-producers spread has always been a serious healthcare obstacle. Therefore, such infectious agents are worthy of all the current research. AmpC β-lactamases-producers are the lesser known members of the β-lactamase producers which are mainly characterized by hyperproduction of chromosomally mediated cephalosporinases. These display their resistance to several antibiotics headlined by most penicillins, in addition to others like cefoxitin, cefazolin, and cephalothin. The plasmid-mediated AmpC producers (pAmpC) were previously shown to display resistance to aminopenicillins, carboxypenicillins, and ureidopenicillins; yet they were susceptible to amdinocillin and temocillin (1). AmpC generation has linked its producers to multidrug resistance (MDRs) which has rendered those bacteria resistant to all β-lactams with the clear exception to some carbapenems and fourth-generation cephalosporins (C4), as an example (1, 2). It is actually their resistance to ESBL inhibitors and ability to hydrolyze cephamycins, what sets them apart from other β-lactamase producers (3).

The AmpC distribution has seen isolates in *Escherichia coli, Klebsiella pneumoniae, Klebsiella oxytoca, Salmonella*, and *Proteus mirabilis* (4); with the former two actually being the most predominant, taking on the endemic and epidemic characteristics, respectively (2, 5). A strong plasmidic spread between different strains of Enterobacteriaceae makes the plasmid-mediated resistance mechanisms dominate this entire species (6, 7). The necessity of AmpC detection mainly allows for the selection of targeted narrow-spectrum antibiotics instead of the risk of working with broad-spectrum antibiotics, thus this will minimize the risk of selection for or promotion of resistance. A Lebanese study showed a high rate of carbapenem resistance among *Acinetobacter* spp. isolates from Saint George's Hospital University Medical Center (SGHUMC), a large tertiary care center in the capital Beirut (8). However, other studies from various Lebanese regions showed low rates of carbapenem resistance with ranges of approximately 60–76% of isolates being reported as such (9, 10). A study from North Lebanon documented the *in-vitro* competition assays in both sensitive and resistant isolates by following up on fitness variations by ESBLs in *E. coli* and *K. pneumoniae* (11). Extended-spectrum β-lactamases-production genes in these two strains might have a fitness cost that lowers the frequency of interspecies competitions by these bacteria. Despite the scarcity of local reports describing β-lactamases efficiently, let alone AmpC among Lebanese patients, some studies from other Arab countries highlight that *K. pneumoniae* strains might actually be the major therapeutic and epidemiological hazard facing such countries—in accordance to a Libyan study (2).

Still, it is vital to distinguish bacterial detection between different hospital wards, since a lot of variances can be deduced when it comes to carriage vs. infection and to the intensive care unit (ICU), as an example. A study involving multiple ICUs at different medical centers in Lebanon exhibited that the overall numbers of ESBL strain carriers and infected were increased by almost two- and three-fold during ICU admission (12). This occurrence seemed to rise 72 h post ICU admission and fall afterwards up to 15 days later (only to increase after that) with the reasoning behind this being the possible overuse of carbapenems in these patients. It is essential then to implement strict hygiene procedures and eliminate any possible risk factors that contribute to β-lactamases-producers' spread—more specifically AmpC (plasmidic and chromosomal), and to deploy regular surveillance studies to determine the genetic basis of resistance.

To our knowledge, medical centers in Lebanon do not separate between ESBL and AmpC. Actually, in most cases, AmpC producers are represented and treated the same as ESBLs; as a result, they are not isolated on wards as entities of their own. Therefore, our aim in this study was to determine the numbers and percentages of AmpC Enterobacteriaceae isolates, notably plasmid-mediated ones, across different wards at the Centre Hospitalier du Nord (CHN), Lebanon, and highlight the importance of infection control protocols.

## Materials and Methods

The study was carried out on the premises of CHN and analyzed on the campus of the University of Balamand. Carriage and infection with pAmpC were retrospectively investigated between 2011 and 2015 and prospectively between 2016 and 2019. The data was inclusive of the patients admitted to the tertiary care center as a representative sample of the Lebanese population, both adults and pediatrics.

### Study Population

The study sample of CHN patients was provided by hospital laboratory staff. The patients were distributed among wards that included: ICU, Cardiac Surgical Unit (CSU), Pediatric Unit, Neonatal Unit, Emergency Department Unit (ED), Infirmary Unit, Outpatient Department Unit (OPD), One Day Surgery Unit, Oncology Unit, and floors 2–5. The latter floors included both surgery and medicine adult patients and were not limited to certain organ system based complaints.

### Samples

Isolates were collected in the form of cultures that included: Blood, central venous lines, urine, urinary catheters, dialysis catheters, feces, rectal swabs, anal swabs, nasal swabs, pus, abscesses, surgical drains, lacrimal secretions, abdominal ascitic fluids, bronchoalveolar lavage, and chemotherapy chambers. The samples were selected for all patients of any age and gender groups. Patients who had negative culture growth were excluded. In addition, only one carriage sample was selected per patient for those that presented multiple times with the same organism, same culture type, and same antibiogram. There was a resultant total of 419 AmpC producers. The isolates' culture growth was achieved through the microbiology labs of CHN.

### Phenotypic Detection of AmpC and Genotypic Detection of pAmpC Genes

#### Screening for Cefoxitin Resistant Isolates

As a screening method for AmpC production, Cefoxitin disks (30 μg) were used (13). Inhibition zones smaller than 18 mm were considered as indicators of potential AmpC production and these strains were subjected to further confirmatory testing.

#### Confirmatory Tests for AmpC β-Lactamase

Using Cefoxitin-cloxacillin double disk synergy test (13), and AmpC induction tests (14). *Klebsiella pneumonia*e strain (M40) was used as the positive control strain for all the tests. Briefly, the Cefoxitin-cloxacillin double disk synergy test was performed on all the potentially AmpC producing isolates. These were grown on Mueller Hinton agar (Oxoid, USA). Cefoxitin/cloxacillin disks (30 μg) and Cefoxitin disk (30 μg) were used to determine the inhibition zone difference. A minimum diameter of 4 mm of inhibition of Cefoxitin/cloxacillin over Cefoxitin alone was considered a confirmation of AmpC production (13).

In this study, all patients admitted were screened for the fecal carriage of AmpC. Preliminary screening was done by inoculating stools on MacConckey agar supplemented with 2 μg/ml of Cefotaxime (bioMérieux, La Balme-les-Grottes, France). Suspect colonies growing after 24–48 h of incubation were subject to phenotypic and genotypic identification of pAmpC. We defined “Infected” any patient who had an infection in any part of the body that grew an AmpC producer regardless if an AmpC producer was isolated from the stools of this patient. We defined “Carrier” any patient from whom an AmpC producer was isolated (stools screen) without a documentation of infection in this patient.

### Molecular Detection of pAmpC

Plasmid DNA extraction was done using the Qiagen Plasmid Purification Midi Kits (Qiagen, Courtaboeuf, France). All the bacteria that were isolated before 2015 were kept at −70°C after DNA extraction and purification. The isolates of 2015 and beyond were subject to prospective PCR experiments. Six different gene families of pAmpC β-lactamases were investigated according to Pérez-Pérez and Hanson (15), these were: ACC, CIT, DHA, EBC, FOX, and MOX. All primers were synthesized and supplied by Bio-Rad (Germany).

Two annealing temperatures were used including 54°C for amplification of genes belonging to the FOX and MOX families and 64°C for the other four genes families.

MOXMF: 5′ GCT GCT CAA GGA GCA CAG GAT 3′
MOXMR: 5′ CAC ATT GAC ATA GGT GTG GTG C 3′
CITMF: 5′ TGG CCA GAA CTG ACA GGC AAA 3′
CITMR: 5′ TTT CTC CTG AAC GTG GCT GGC 3′
DHAMF: 5′ AAC TTT CAC AGG TGT GCT GGG T 3′
DHAMR: 5′ CCG TAC GCA TAC TGG CTT TGC 3′
ACCMF: 5′ AAC AGC CTC AGC AGC CGG TTA 3′
ACCMR: 5′ TC GCC GCA TC ATC CCT AGC 3′
EBCMF: 5′ TCG GTA AAG CCG ATG TTG CGG 3′
EBCMR: 5′ CTT CCA CTG CGG CTG CAA GTT 3′
FOXMR: 5′ AAC ATG GGG TAT CAG GGA GAT G 3′
FOXMR: 5′ CAA AGC GCG TAA CCG GAT TGG 3′.

A single bacterial colony of each isolate was inoculated into 5 ml of Luria-Bertani broth (Difco, Detroit, Mich.) and incubated overnight at 37°C. A bacterial pellet was obtained after centrifugation (5,000 g for 20 min). The pellet was re-suspended in sterile distilled water and bacterial cells were lysed by heating at 95°C for 10 min followed by high speed centrifugation. PCR was performed according to Pérez-Pérez and Hanson (15) and PCR product were analyzed by gel electrophoresis with 2% agarose (Bio-Rad, Hercules, Calif.) and ethidium bromide at 10 μg/ml and visualized by UV transillumination.

Isolates that did not amplify at least one of the above genes were not considered pAmpC producers and were not therefore included in this study.

### Statistical Analysis

The provided data was analyzed using both the Statistical Package for Social Sciences (SPSS) and Microsoft Excel. The resultant pAmpC were divided with respect to annual distribution of infection vs. carriage states. These states were consequently divided as per hospital wards. The percentage displayed at each ward was the number of isolates detected in accordance to the total number of infection or carriage strains. Each was calculated as: Number of isolates in a specific ward/Total Number of isolates.

### Infection Control Policies and Procedures

The policies followed by the Infection Control Department at CHN were in parallel to those initiated by the Centers for Disease Control and Prevention (CDC) (16). These policies were constituted from both standard and transmission-based precautions. The implementation of standard precautions was the primary strategy to prevent any transmissions locally between healthcare personnel which housed the following elements: Hand and respiratory hygiene, use of protective equipment (gloves, masks…etc.), sterile instrumentation and injection practices, disinfected surfaces, and safe disposal of sharps. Transmission-based precautions were applied for patients with confirmed or suspected communicable infections; these included droplet, airborne, and contact precautions. Protective isolation was mainly used for oncology patients; these patients were placed in rooms with high-efficiency particulate air (HEPA) filtration air and positive air room pressure relative to the outside. Note that when the transmission-based precautions and/or the protective isolation were used alone or as a combination, the application of standard precautions was indispensable regardless.

The patients transferred to CHN from an outside facility where he/she has stayed ≥72 h, were placed in contact isolation until all cultures were done and the results were available. The patients that were admitted or transferred to the ICU were placed in contact isolation rooms until the results of the blood, urine, and sputum cultures were available. When possible, a single room is indicated for the following: (A) Patients with highly transmissible or epidemiologically important microorganisms [vancomycin-resistant enterococci (VRE), methicillin-resistant *Staphylococcus aureus* (MRSA), tuberculosis (Tb), chickenpox, and respiratory syncytial virus (RSV)], (B) patients with poor hygiene habits and those who cannot achieve best assistance in maintaining the infection control precautions, and (C) as deemed by an infectious disease specialist or infection control team. Centre Hospitalier du Nord reported strict application of the policies by 2014.

## Results

The Annual Distribution of the pAmpC isolates with respect to the states of infection and carriage according to the different hospital units from 2011 to 2019 is shown in Table 1. The highest infection percentage was 50.00% (a tie with the non-critical care) in 2011 with only two isolates. The highest infection isolates' number were in 2012 and 2013 (nine in both years; 22.50 and 20.00%, respectively). The number of carriage isolates was the highest in 2013 (10 isolates with 28.57%). Intensive care unit pAmpC carriage data initially exhibited a rise from a single isolate to seven over a 3-year period only to then decrease to zero across a 4-year period to 2018 matching the recorded isolation protocol at CHN; however, one isolate was documented in 2019 (Table 2A). Infection isolates rose from one to seven in 2014 only to decrease afterwards reaching a single isolate in 2019.

**Table 1:** The annual distribution of the pAmpC isolates with respect to the states of infection and carriage in accordance to different hospital units from 2011 to 2019.

		**Total** **pAmpC**	**Critical care** **units**		**Non-critical care** **adult wards unit**		**Outpatients unit**		**One day** **surgery unit**		**Non-adult** **wards unit**	
**Years**	**Carrier/** **Infected**	**Total** **isolates**	**Nb**	**%**	**Nb**	**%**	**Nb**	**%**	**Nb**	**%**	**Nb**	**%**
2011	Carrier	9	1	11.11	4	44.44	2	22.22	1	11.11	1	11.11
	Infected	4	2	50.00	2	50.00	0	0.00	0	0.00	0	0.00
2012	Carrier	17	6	35.30	6	35.30	1	5.88	1	5.88	3	17.65
	Infected	40	9	22.50	20	50.00	1	2.50	4	10.00	6	15.00
2013	Carrier	35	10	28.57	14	40.00	5	14.29	4	11.43	2	5.71
	Infected	45	9	20.00	30	66.67	2	4.44	0	0.00	4	8.89
2014^*^	Carrier	18	5	27.78	13	72.22	0	0.00	0	0.00	0	0.00
	Infected	33	7	21.21	23	69.70	0	0.00	0	0.00	3	9.09
2015	Carrier	18	4	22.22	14	77.78	0	0.00	0	0.00	0	0.00
	Infected	28	4	14.29	17	60.71	1	3.57	1	3.57	5	17.86
2016	Carrier	21	2	9.52	13	61.90	5	23.81	0	0.00	1	4.76
	Infected	58	1	1.72	37	63.79	14	24.14	0	0.00	6	10.34
2017	Carrier	14	2	14.29	8	57.14	4	28.57	0	0.00	0	0.00
	Infected	29	0	0.00	10	34.48	15	51.72	0	0.00	4	13.79
2018	Carrier	14	1	7.14	6	42.86	6	42.86	0	0.00	1	7.14
	Infected	13	2	15.38	4	30.76	5	38.46	1	7.69	1	7.69
2019	Carrier	10	3	30.00	2	20.00	4	40.00	0	0.00	1	10.00
	Infected	13	1	7.69	4	30.77	8	61.54	0	0.00	0	00.00

*1. Critical care units: ICU + CSU. 2. Non-critical care adult wards unit: floors 2, 3, 4, and 5 + oncology. 3. Outpatients unit: outpatient department + emergency department (ED) + infirmary. 4. One day surgery unit. 5. Non-adult wards unit: pediatrics + neonatal. Nb, number of isolates; %, Percentage: (Number of isolates in a specific ward/Total Number of isolates) ×100*.

* *2014: The start of the infection control policies*.

**Table 2A:** The annual distribution of the pAmpC isolates with respect to the states of infection and carriage according to the hospital's ICU and CSU ward data from 2011 to 2019.

		**ICU**		**CSU**	
**Years**	**Carrier/Infected**	**Nb**	**%**	**Nb**	**%**
2011	Carrier	1	11.11	0	0.00
	Infected	1	25.00	1	25.00
2012	Carrier	4	23.53	2	11.76
	Infected	5	12.50	4	10.00
2013	Carrier	7	20.00	3	8.57
	Infected	5	11.11	4	8.89
2014^*^	Carrier	5	27.78	0	0.00
	Infected	7	21.21	0	0.00
2015	Carrier	4	22.22	0	0.00
	Infected	3	10.71	1	3.57
2016	Carrier	0	0.00	2	9.52
	Infected	1	1.72	0	0.00
2017	Carrier	2	14.29	0	0.00
	Infected	0	0.00	0	0.00
2018	Carrier	0	0.00	1	7.14
	Infected	1	7.69	1	7.69
2019	Carrier	1	10.00	2	20.00
	Infected	1	7.69	0	0.00

*Nb, number of isolates; Nb, Number of isolates. %, percentage: (number of isolates in a specific ward/total number of isolates) ×100. One day surgery was not included in Table 2.*

* *2014: The start of the infection control policies*.

The non-critical care adult wards units dominated the majority of the numbers and percentages for both of the infection and carriage categories from each year. The highest non-critical care carriage percentage was 77.78% in 2015 and 69.70% for infection in 2014. Both would reach their lowest recorded percentage by 2019 with 20.00% for carriage and 30.77% for infection. When the non-critical care adult wards were looked at individually (Table 2B), floors 2, 3, and 4 seemed to have the highest share of infection and carriage with floor 4 showing a decrease in both categories by 2019 reaching zero isolates as compared to two and one, respectively, in 2011. Such a decrease in isolates came after the high number of isolates for infection and carriage that seemed to persist from 2013 to 2016. The highest number of infection and carriage isolates for floor 4 were 16 in 2016 and six in 2015, respectively. Floor 3 had one carriage isolate in 2011 with zero infection as compared to 2019 with zero carriage and two isolates in the infection category. As for the highest number of infection and carriage isolates, they were 14 in 2016 and seven in 2014, respectively. The year 2016 for floor 2 had its highest number of infection and carriage isolates with seven in 2014 and 2016 and five in 2016, respectively. Floor 5 documented one isolate for infection recorded in 2019 coming off of a 4-year streak of nil values from 2015. That was on the back of six infection isolates in 2013. A single carriage isolate was documented in 2013 and 2018. The oncology floor showed six infection isolates in 2013, two in 2013, and one in 2015 with the rest of the years being nil up to 2019. Carriage reached a maximum of four isolates in 2013 with the rest being nil values afterwards with exception to singles isolates each in 2016 and 2019.

**Table 2B:** The annual distribution of the pAmpC isolates with respect to the states of infection and carriage according to the hospital's floors 2, 3, 4, and 5 and the oncology ward data from 2011 to 2019.

		**Floor 2**		**Floor 3**		**Floor 4**		**Floor 5**		**Oncology**	
**Years**	**Carrier/Infected**	**Nb**	**%**	**Nb**	**%**	**Nb**	**%**	**Nb**	**%**	**Nb**	**%**
2011	Carrier	1	11.11	1	11.11	2	22.22	0	0.00	0	0.00
	Infected	0	0.00	0	0.00	1	25.00	1	25.00	0	0.00
2012	Carrier	1	5.88	1	5.88	2	11.76	0	0.00	2	11.76
	Infected	2	5.00	4	10.00	5	12.50	3	7.50	6	15.00
2013	Carrier	2	5.71	3	8.57	4	11.43	1	2.86	4	11.43
	Infected	6	13.33	9	20.00	7	15.56	6	13.33	2	4.44
2014^*^	Carrier	2	11.11	7	38.89	4	22.22	0	0.00	0	0.00
	Infected	7	21.21	7	21.21	8	24.24	1	3.03	0	0.00
2015	Carrier	3	16.67	5	27.78	6	33.33	0	0.00	0	0.00
	Infected	4	14.29	6	21.43	6	21.43	0	0.00	1	3.57
2016	Carrier	5	23.81	4	19.05	3	14.29	0	0.00	1	4.76
	Infected	7	12.07	14	24.14	16	27.59	0	0.00	0	0.00
2017	Carrier	3	21.43	2	14.29	3	21.43	0	0.00	0	0.00
	Infected	2	6.90	4	13.79	4	13.79	0	0.00	0	0.00
2018	Carrier	1	7.14	2	14.29	2	14.29	1	7.14	0	0.00
	Infected	0	0.00	1	7.69	3	23.08	0	0.00	0	0.00
2019	Carrier	1	10.00	0	0.00	0	0.00	0	0.00	1	10.00
	Infected	1	7.69	2	15.38	0	0.00	1	7.69	0	0.00

*Nb, number of isolates; Nb, Number of isolates. %, percentage: (number of isolates in a specific ward/total number of isolates) ×100. One day surgery was not included in Table 2*.

* *2014: The start of the infection control policies*.

The outpatients unit had a low number of isolates in both categories up until 2016 with five (23.81%) and 14 (24.14%) for carriage and infection, respectively (Table 1). Infection overtook the non-critical care adult wards unit's top spot from 2017 to 2019 with 15 isolates (51.72%) to eight isolates (61.54%), respectively. Outpatients unit's carriage tied with the non-critical care one in 2018 with six isolates each (42.86%) and took the top spot in 2019 with four isolates (40.00%). Individually, a rise in pAmpC carriage and infection isolates was noted in the Outpatients Department notably in 2016 onwards with the highest number of isolates reaching nine in 2017 and four in 2018 for the infection and carriage, respectively (Table 2C). The infirmary had a maximum of five isolates for infection in 2016 and three isolates in both 2016 and 2017. The ED did not exceed two isolates for carriage (2013 and 2018) and three isolates for infection (2016 and 2017).

**Table 2C:** The annual distribution of the pAmpC isolates with respect to the states of infection and carriage according to the hospital's pediatric, outpatients, infirmary, ED, and neonatal ward data from 2011 to 2019.

		**Pediatric** **floor**		**Outpatient** **department**		**Infirmary**		**ED**		**Neonatal**	
**Years**	**Carrier/Infected**	**Nb**	**%**	**Nb**	**%**	**Nb**	**%**	**Nb**	**%**	**Nb**	**%**
2011	Carrier	0	0.00	1	11.11	0	0.00	1	11.11	1	11.11
	Infected	0	0.00	0	0.00	0	0.00	0	0.00	0	0.00
2012	Carrier	1	5.88	1	5.88	0	0.00	0	0.00	2	11.76
	Infected	4	10.00	0	0.00	1	2.50	0	0.00	2	5.00
2013	Carrier	0	0.00	2	5.71	1	2.86	2	5.71	2	5.71
	Infected	1	2.22	2	4.44	0	0.00	0	0.00	3	6.67
2014^*^	Carrier	0	0.00	0	0.00	0	0.00	0	0.00	0	0.00
	Infected	2	6.06	0	0.00	0	0.00	0	0.00	1	3.03
2015	Carrier	0	0.00	0	0.00	0	0.00	0	0.00	0	0.00
	Infected	2	7.14	0	0.00	0	0.00	1	3.57	3	10.71
2016	Carrier	1	4.76	1	4.76	3	14.29	1	4.76	0	0.00
	Infected	2	3.45	6	10.34	5	8.62	3	5.17	4	6.90
2017	Carrier	0	0.00	2	14.29	2	14.29	0	0.00	0	0.00
	Infected	2	6.90	9	31.03	3	10.34	3	10.34	2	6.90
2018	Carrier	1	7.14	4	28.57	0	0.00	2	14.29	0	0.00
	Infected	1	7.69	5	38.46	0	0.00	0	0.00	0	0.00
2019	Carrier	1	10.00	3	30.00	1	10.00	0	0.00	0	0.00
	Infected	0	0.00	7	53.85	0	0.00	1	7.69	0	0.00

*Nb, number of isolates; Nb, Number of isolates. %, percentage: (number of isolates in a specific ward/total number of isolates) ×100. One day surgery was not included in Table 2*.

* *2014: The start of the infection control policies*.

One day surgery unit had a maximum of four infection isolates in 2012 and four carriage isolates in 2013 (Table 1). For the non-adult wards unit, infection isolates were at a maximum of six 2012 and 2016 (15.00 and 10.34%, respectively) while carriage isolates had a maximum of only two isolates in 2013 (5.71%). The pediatric floor showed four infection isolates in 2012 with other numbers ranging between nil and two isolates across the other years. Carriage never exceeded one isolate overall. The neonatal floor had a maximum of four infection isolates in 2016 with carriage being nil mostly with the highest of two isolates in 2012 and 2013 (Table 2C).

**Figure 1:**
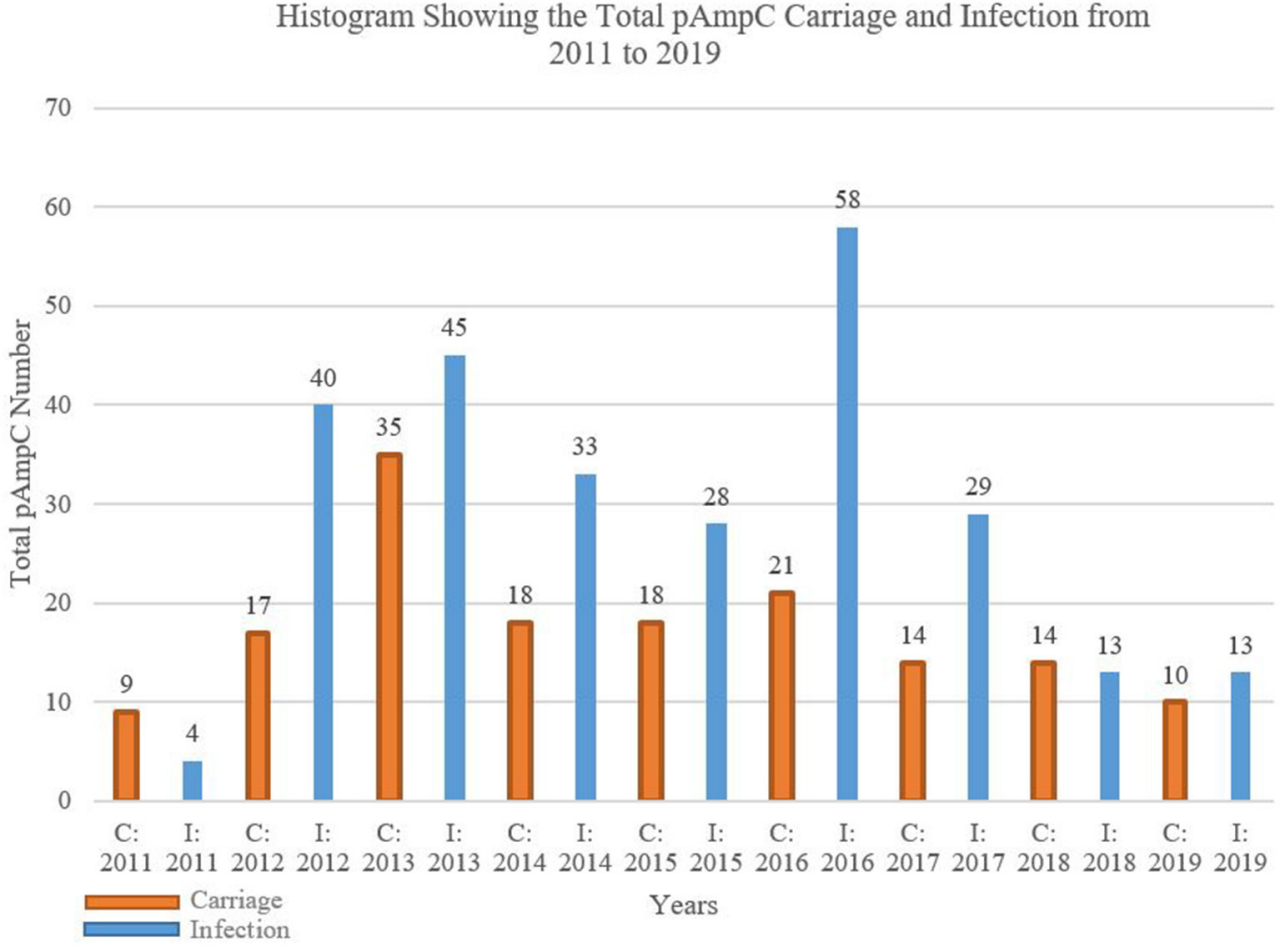
Histogram showing the total pAmpC carriage and infection from 2011 to 2019. C, carriage; I, infection.

The overall AmpC infection exceeded that of carriage with the mere exceptions to 2011 and 2018, for which carriage surpasses infection by nine to four and 14 to 13 isolates, respectively (Figure 1). The biggest gap between the two categories was in 2016 with 58 infected isolates to the carriage's 21.

The FOX gene was most prevalently detected by PCR when it came to pAmpC expression (Table 3). FOX dominated the carriage and infection categories throughout the entire 9-year period with the highest PCR detection happening in 2013 for carriage and 2016 for infection. The FOX percentage was the highest for carriage in 2015 with 83.33% (18 total isolates) and for infected in 2011 with 75% (four total isolates). PCR detection of EBC was nil at all times; on the other hand, the relative fall in PCR detection in the last 2 years was evident for the other five genes.

**Table 3:** The annual distribution of the six gene families of pAmpC isolates with respect to the states of infection and carriage from 2011 to 2019.

		**The six investigated gene families of pAmpC**												
**Years**	**Carriage/Infection**	**Total isolates**	**MOX**		**CIT**		**DHA**		**ACC**		**EBC**		**FOX**	
			**Nb**	**%**	**Nb**	**%**	**Nb**	**%**	**Nb**	**%**	**Nb**	**%**	**Nb**	**%**
2011	Carrier	9	1	11.11	0	0.00	1	11.11	1	11.11	0	0.00	6	66.67
	Infected	4	1	25.00	0	0.00	0	0.00	0	0.00	0	0.00	3	75.00
2012	Carrier	17	4	23.53	3	17.65	0	0.00	2	11.76	0	0.00	8	47.06
	Infected	40	11	27.50	4	10.00	8	20.00	1	2.50	0	0.00	16	40.00
2013	Carrier	35	9	25.71	1	2.86	3	8.57	0	0.00	0	0.00	22	62.86
	Infected	45	11	24.44	1	2.22	10	22.22	0	0.00	0	0.00	23	51.11
2014^*^	Carrier	18	4	22.22	1	5.56	2	11.11	1	5.56	0	0.00	10	55.56
	Infected	33	6	18.18	1	3.03	1	3.03	3	9.09	0	0.00	22	66.67
2015	Carrier	18	2	11.11	0	0.00	0	0.00	1	5.56	0	0.00	15	83.33
	Infected	28	5	17.86	4	14.29	4	14.29	3	10.71	0	0.00	12	42.86
2016	Carrier	21	2	9.52	2	9.52	1	4.76	0	0.00	0	0.00	16	76.19
	Infected	58	9	15.52	8	13.79	5	8.62	1	1.72	0	0.00	35	60.34
2017	Carrier	14	4	28.57	0	0.00	0	0.00	0	0.00	0	0.00	10	71.43
	Infected	29	8	27.59	0	0.00	0	0.00	1	3.45	0	0.00	20	68.97
2018	Carrier	14	3	21.43	0	0.00	0	0.00	0	0.00	0	0.00	11	78.57
	Infected	13	5	38.46	2	15.38	2	15.38	0	0.00	0	0.00	4	30.77
2019	Carrier	10	2	20.00	0	0.00	0	0.00	0	0.00	0	0.00	8	80.00
	Infected	13	5	38.46	0	0.00	0	0.00	0	0.00	0	0.00	8	61.54

*Nb, number of isolates; %, percentage: (number of isolates in a specific ward/total number of isolates) ×100*.

* *2014: The start of the infection control policies*.

Despite the infection control policies implications in 2014, the total pAmpC numbers for carriage and infection only started to notably decrease in 2017 (Table 4). *Escherichia coli* pAmpC seemed to dominate over *K. pneumoniae* and *K. oxytoca* for both carriage and infection states across the years. *Escherichia coli* numbers were highest in 2013 with 25 isolates for carriage and in 2016 with 41 for infection. The ICU isolates were also predominately *E. coli* which seemed to decrease post 2014. Floors 2–5 housed the highest number of isolates in most of the years with 2013 and 2016 being the most notable especially for floor 3 that exhibited pAmpC isolates of the three mentioned bacteria for the infection category; *K. oxytoca* was only present in these two occasions. *Klebsiella pneumoniae* pAmpC isolates were highest in 2012 and 2013 for infection with 15 isolates each, and in 2013 for carriage with 10 isolates.

**Table 4:** The annual distribution of the total pAmpC isolates with respect to species and the states of infection and carriage in accordance to different hospital units from 2011 to 2019.

**Year**	**2011**	**2011**	**2012**	**2012**	**2013**	**2013**	2014^*^	2014^*^	**2015**	**2015**	**2016**	**2016**	**2017**	**2017**	**2018**	**2018**	**2019**	**2019**
Total pAmpC	4	9	40	17	45	35	33	18	28	18	58	21	29	14	13	14	13	13
*E. coli*	3	8	25	11	28	25	26	15	27	16	41	17	25	11	12	12	10	10
*K. pneumoniae*	1	1	15	6	15	10	7	3	1	2	14	4	4	3	1	2	3	3
*K. oxytoca*	0	0	0	0	2	0	0	0	0	0	3	0	0	0	0	0	0	0
ICU	1.0.0	1.0.0	3.2.0	3.1.0	5.0.0	5.2.0	6.1.0	5.0.0	3.0.0	3.1.0	1.0.0	0	0	2.0.0	0.1.0	0	1.0.0	1.0.0
CSU	1.0.0	0	3.1.0	2.0.0	2.2.0	1.1.0	0	0	0.1.0	0	0	1.1.0	0	0	1.0.0	1.0.0	0	0
Neonatal	0	1.0.0	2.0.0	2.0.0	3.0.0	2.0.0	1.0.0	0	3.0.0	0	4.0.0	0	2.0.0	0	0	0	0	0
Floor 2	0	1.0.0	2.0.0	0.1.0	3.3.0	2.0.0	4.3.0	1.1.0	4.0.0	3.0.0	5.2.0	5.0.0	2.0.0	3.0.0	0	0.1.0	1.0.0	1.0.0
Floor 3	0	0.1.0.	2.2.0	1.0.0	6.2.1	2.1.0	7.0.0	5.2.0	6.0.0	4.1.0	9.4.1	2.2.0	2.2.0	2.0.0	1.0.0	2.0.0	2.0.0	2.0.0
Floor 4	0.1.0	2.0.0.	4.1.0	1.1.0	5.2.0	2.2.0	4.3.0	4.0.0	6.0.0	6.0.0	9.7.0	3.0.0	4.0.0	1.2.0	3.0.0	2.0.0	0	0
Floor 5	1.0.0	0	3.0.0	0	5.1.0	1.0.0	2.0.0	0	0	0	0	0	0	0	0	1.0.0	1.0.0	1.0.0
Pediatric floor	0	0	2.2.0	0.1.0	1.0.0	0	2.0.0	0	2.0.0	0	2.0.0	1.0.0	2.0.0	0	1.0.0	1.0.0	0	0
Outpatiwnt department	0	1.0.0	0	0.1.0	0.2.0	1.1.0	0	0	0	0	6.0.0	1.0.0	7.2.0	2.0.0	5.0.0	3.1.0	4.3.0	4.3.0
Infirmary	0	0	0.1.0	0	0	1.0.0	0	0	0	0	5.0.0	2.1.0	2.0.0	1.1.0	0	0	0	0
Oncology	0	0	2.4.0	1.1.0	0.2.0	2.2.0	0	0	1.0.0	0	0	1.0.0	0	0	0	0	0	0
ED	0	1.0.0	0	0	0	2.0.0	0	0	1.0.0	0	3.0.0	1.0.0	3.0.0	0	0	2.0.0	1.0.0	1.0.0
One day surgery unit	0	1.0.0	2.2.0	0.1.0	0	3.1.0	0	0	1.0.0	0	0	0	0	0	1.0.0	0	0	0
C/I	I	C	I	C	I	C	I	C	I	C	I	C	I	C	I	C	I	I

*C, carriage; I, infection*.

* *2014: the start of the infection control policies*.

The total number of strains seemed to relatively increase with the years despite the initiation of the infection control measures (Tables 5A–C). *Escherichia coli* was the dominant strain with a total number of 1,004 by 2019 that spread across multiple wards. The outpatient department had the highest numbers from 2017 onwards; floor 5 was in second place in terms of the numbers (Table 5A). Both Floor 5 and the outpatient department had the highest numbers of *K. pneumoniae* strains by over a 3-year period from 2017 (Table 5B). Involvement of both wards seemed to carry on as well for *K. oxytoca* (Table 5C). The ICU was never spared from all three organisms as it always included a number of all three strains by 2019.

**Table 5A:** The annual distribution of the total *E. coli* isolates in accordance to different hospital units from 2011 to 2019.

**Year**	***E. coli***	**ICU**	**CSU**	**Neonatal**	**Floor 2**	**Floor 3**	**Floor 4**	**Floor 5**	**Pediatric** **floor**	**Outpatient** **department**	**Infirmary**	**Oncology**	**ED**	**One day** **surgery unit**
2011	692	NA	NA	NA	NA	NA	NA	NA	NA	NA	NA	NA	NA	NA
2012	760	NA	NA	NA	NA	NA	NA	NA	NA	NA	NA	NA	NA	NA
2013	899	NA	NA	NA	NA	NA	NA	NA	NA	NA	NA	NA	NA	NA
2014^*^	849	NA	NA	NA	NA	NA	NA	NA	NA	NA	NA	NA	NA	NA
2015	972	NA	NA	NA	NA	NA	NA	NA	NA	NA	NA	NA	NA	NA
2016	1004	NA	NA	NA	NA	NA	NA	NA	NA	NA	NA	NA	NA	NA
2017	946	52	15	26	71	65	72	131	39	267	22	61	87	38
2018	1001	28	21	16	83	109	65	201	89	302	11	59	17	0
2019	1004	69	29	11	99	78	59	198	91	228	8	57	59	18

*NA, not applicable*.

* *2014: The start of the infection control policies*.

**Table 5B:** The annual distribution of the total *K. pneumoniae* isolates in accordance to different hospital units from 2011 to 2019.

**Year**	***K. pneumoniae***	**ICU**	**CSU**	**Neonatal**	**Floor 2**	**Floor 3**	**Floor 4**	**Floor 5**	**Pediatric** **floor**	**Outpatient** **department**	**Infirmary**	**Oncology**	**ED**	**One day** **surgery unit**
2011	122	NA	NA	NA	NA	NA	NA	NA	NA	NA	NA	NA	NA	NA
2012	130	NA	NA	NA	NA	NA	NA	NA	NA	NA	NA	NA	NA	NA
2013	117	NA	NA	NA	NA	NA	NA	NA	NA	NA	NA	NA	NA	NA
2014^*^	201	NA	NA	NA	NA	NA	NA	NA	NA	NA	NA	NA	NA	NA
2015	168	NA	NA	NA	NA	NA	NA	NA	NA	NA	NA	NA	NA	NA
2016	211	NA	NA	NA	NA	NA	NA	NA	NA	NA	NA	NA	NA	NA
2017	199	12	5	2	18	22	11	38	16	56	10	2	5	2
2018	218	14	7	4	11	28	24	46	18	43	8	8	3	4
2019	221	19	7	3	19	16	19	44	16	38	17	5	11	7

*NA, not applicable*.

* *2014: The start of the infection control policies*.

**Table 5C:** The annual distribution of the total *K. oxytoca* isolates in accordance to different hospital units from 2011 to 2019.

**Year**	***K. oxytoca***	**ICU**	**CSU**	**Neonatal**	**Floor 2**	**Floor 3**	**Floor 4**	**Floor 5**	**Pediatric** **floor**	**Outpatient** **department**	**Infirmary**	**Oncology**	**ED**	**One day** **surgery unit**
2011	15	NA	NA	NA	NA	NA	NA	NA	NA	NA	NA	NA	NA	NA
2012	22	NA	NA	NA	NA	NA	NA	NA	NA	NA	NA	NA	NA	NA
2013	26	NA	NA	NA	NA	NA	NA	NA	NA	NA	NA	NA	NA	NA
2014^*^	13	NA	NA	NA	NA	NA	NA	NA	NA	NA	NA	NA	NA	NA
2015	24	NA	NA	NA	NA	NA	NA	NA	NA	NA	NA	NA	NA	NA
2016	16	NA	NA	NA	NA	NA	NA	NA	NA	NA	NA	NA	NA	NA
2017	17	0	0	0	2	1	1	8	1	4	0	0	0	0
2018	23	0	0	0	4	2	1	11	0	5	0	0	0	0
2019	28	19	7	3	19	16	19	44	16	38	17	5	11	7

*NA, not applicable*.

* *2014: The start of the infection control policies*.

**Figure 2:**
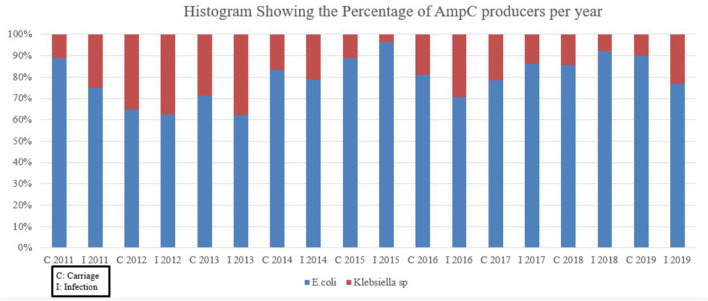
Histogram showing the percentage of pAmpC procedure per year.

Figure 2 does not take into account pAmpC in specifics but rather deals with AmpC producers as a whole. *Escherichia coli* was the dominant AmpC producer across the years with percentages exceeding 60% over the 8-year period. After the alleged 2014 implementation of isolation protocols, the percentage of AmpC by *E. coli* remained high while *Klebseilla* sp reached a maximum of approximately 30% in 2016 for the infection group.

## Discussion

Like any other medical institutes worldwide, Lebanese healthcare centers are susceptible to bacterial spread. Although regarded inferior to ESBLs, as in a topic of research, AmpC β-lactamase producers' spread is just as serious. One study that focused on the occurrences and the mechanisms of resistance of MDR Gram-negative bacilli in some Lebanese hospitals' sewage treatment plants noted a detection of AmpC producers of 25 and 28.9% of all isolates in two hospitals (17).

### The Molecular Methods of Resistance

Resistances toward a β-lactam antibiotic in Gram-negative bacteria can be conferred through three main levels to prevent cellular death of the bacterium. First, a diffusion through the outer cellular membrane can be halted by the blockage of porins and the efflux pump. Second, the strong work of the β-lactamases prevents the diffusion through the peptidoglycan cell wall. Third, mutations in the penicillin binding proteins (PBP) would result in failure to bind to the β-lactam antibiotic and thus preventing cellular demise.

AmpC is highlighted through the actions of AmpD—an amidase which participates in recycling of peptidoglycan—and AmpR—a transcriptional regulator which represses the expression of AmpC (18). For the wild type/uninduced AmpC, AmpD recycles the cell wall fragments and AmpR plays its role as a repressor; therefore, the AmpC β-lactamase gene is not expressed. As for high-level production of the enzyme, this can be inducible or constitutive. With inducible production, the enzyme is produced at low levels unless the organism is exposed to the inducing agents, then more recycling would be required from AmpD which becomes overwhelmed and AmpR being converted to an activator. AmpC gene as a result would be expressed. Constitutive production involves inner mutations, with AmpD gene being inactivated and AmpR constantly being converted to a fast-acting activator. Therefore, this results in the hyperexpression of the AmpC gene. The AmpC hyperproduction can indeed happen without an outside inducing agent; this mediates resistance to many β-lactams.

The pAmpCs are encoded on large plasmids and carry additional resistance genes making the producers more potent. As the AmpC gene is regulated by a weak promoter vs. a strong attenuator, diminished levels of many β-lactamases are produced. Therefore, in addition to the chromosomal gene encoding, for AmpC β-lactamases, strains of *E. coli*—which were once cefoxitin susceptible as an example—would gain the pAmpC β-lactamase, the CMY-2, becoming then cefoxitin resistant (19). The FOX family gene is considered a highly prevelant AmpC gene (20). Our data showed a near total dominance of the FOX gene in both carraige and infection with MOX coming in second (Table 3). Other studies also concur with this finding (21, 22), with one exhibiting a 37.2% of *E. coli* isolates expressing FOX-1, and another showing 40.9% of a 22 AmpC genes belonging to the FOX and MOX families (23, 24).

### The Spread Across the Hospital Wards

The spread of AmpC β-lactamases in healthcare centers can have very serious complications (25). Almost all hospital wards are not spared from such a calamity, and the ICU is not an exception. Such Enterobacteriaceae—if not controlled for efficiently—would surely serve in the deterioration of critically ill patients. The critical care units were mainly second when it came to the number of isolates according to our study with the ICU taking the major share (Tables 1, 2A). Our data recorded a rise in pAmpC carriage in the ICU from 1 to 7 isolates (which is the highest number recorded across all wards individually) from 2011 to 2013 (Table 2A), a figure that is of significance given the supposed rarity of AmpC. The results of the implementation of strict isolation protocols at CHN are noted with the decrease afterwards and never returning to the 2013 isolates. This is somewhat mirrored in the pAmpC infection isolates across the 8-year period with the peak number being detected in the same year of the implementation of the strict isolation protocols (Table 2A). Infection control might seem straightforward in terms of appropriate hygiene and prevention of overexposed usage of broad-spectrum antibiotics (19), but other key factors requiring better attention might actually be the prolonged central venous catheterization, indwelling urinary catheters, and intubation/uncoordinated weaning off ventilation attempts; in addition to the long intervals in between diaper changes. These can increase the patients' ICU stay which puts them at higher risk of further infections. Therefore, it is the mere application of the infection control objectives that should be stressed upon. Our study showed that the application of the infection control policies did indeed lead to a drop in the number of isolates in the critical care setting. This can also be attributed to the highly controlled setting of such units due to the critical state of the patients and the more strict measures that might be imposed by the infection control personnel (if found), the attending ICU physician, the senior resident, and/or the nursing supervisor. It is possible that the one nurse to 1–2 patient care setting might play a role in a more focused approach to the implementation of the infection control policies.

The majority of the isolates recovered from Lebanese Medical Centers' ICU were *E. coli* followed by *K. pneumoniae*; which complements our findings at CHN (Figure 2). This is of no surprise when it comes to Lebanese data as it is in agreement with previous local studies and some international research (2, 4, 26). Others postulate that *Pseudomonas* spp. tend to be the most prevalent Gram-negative agents when it comes to ICU infected patients (14, 27). They mainly present as part of respiratory tract infections be it most likely due to prolonged patient intubation or recurrent hospitalizations of immunocompromised patients, with other manifestations ranging from bacteremia to complicated urinary tract infections (UTI) and others. A study from India of 150 ICU patients—in one hospital—had a total number 160 isolates with 35 of them being *Pseudomonas* spp. (14). Out of this number, 51.4% were AmpC. Factors like age >50 years, prolonged endotracheal intubation, extended ICU stay >15 days, and health comorbidities as in hypertension and chronic obstructive pulmonary disease (COPD) were all significant in increasing the risk of ICU pseudomonal infections. The very fact that the 1 day surgery unit had predominantly near-nil AmpC infection and carriage isolate numbers across the years (Table 2C), can show that prolonged hospital stay is a decisive factor for higher risk of infection. This is also reflected in the low number of isolates seen in the ED data. With exception of 2016 and 2019, the oncology unit has maintained almost zero infection and carriage post the infection control protocols; this helps to possibly suggest that the protocols were strictly followed by the healthcare personnel on that ward most likely to avoid jeopardizing the immunocompromised state of such patients.

It is worth to note that the AmpC isolates' numbers from floors 2, 3, and 4 are of approximately close values. A large number of isolates dominated the mentioned floors from 2014 to 2016. Some of these isolates showed even higher numbers post 2014 with values reaching 14 and 16 for floors 3 and 4 in the infection isolates in 2016. Floor 5 was the exception, with it seemingly able to maintain a near zero number of isolates post 2014. This might be down to the fact that floor 5 might have not received a significantly large amount of patients as the other floors. It can also be due to admitting patients that might have not had findings suggestive of an infection or might have not been investigated for carriage. It can also be related to personnel involvement and application of the policies. Such discrepancies would raise the issue of the homogeneity in the implementation of the protocols and actual supervision. It is critical to overcome key hurdles which include a poor sense of staff proficiency, administrative and financial constraints, poorly role-oriented personnel, and subjective negativity in hospital staff attitudes, in order to further succeed in limiting infection spread (28). An efficient implementation of an infection control program can result in an approximate 30% reduction of hospital acquired infections (29). Educational campaigns can play a very important factor in the reduction of infection spread and the promotion of the infection control policies application. The compliance toward at least four elements of the previously mentioned WHO policies seemed to be efficient in reducing respiratory and MDR infections (28, 30). This was previously achieved through the proper education, monitoring and feedback which nearly doubled the percentage of the adherence toward appropriate hand washing technique (31). Therefore, it is the mere application of the infection control objectives that should always be stressed upon.

### Limitations

Our study cannot be looked at without some limitations. Our research was limited to only one center which can diminish the number of the samples provided and subsequently the number of isolates. We plan to consider a future multicenter study that looks more in depth into a larger number of isolates and the trend in both infection and carriage. Our study, however, is hopefully the starting point of future research projects. The alleged tight isolation protocols implemented by CHN are not without flaws since they would still be subject to human error when it comes to strict handling of infection control. Appropriate hygiene through sanitizers' installments, isolation gowns, gloves to the prolonged use of invasive apparatuses, and the overuse of antibiotics, all were not monitored up-close by a research team member. In addition, a suspicion of possible improper specimens' collection and culture analysis might have carried its way into data entry which might explain the sudden nil AmpC values in certain years despite having a significant number of isolates the years before. All of these factors might have contributed to some distortions in the definite linkage between infection control isolation protocols and the numbers of pAmpC isolates. Nevertheless, a relative relationship was detected when these protocols were launched in 2014. As for the data showing the annual distribution of the total bacterial isolates in accordance to different hospital units, a number of missing values can be seen for different hospital wards especially in most of the data predating 2016. This is mainly because of the careful documentation and coordination between the infection control team and hospital wards that was started at that time; it was unfortunate that no detailed records were available before that. However, these tables highlight the relatively high number of total isolates that were detected across the years that almost did not spare any of the wards. When looking at the critical care units, only the adult sections were listed. Therefore, there was not any inclusion of pediatric or neonatal intensive care units which might have had a bulk of infectious microorganisms given the critical setting of the patients. This is especially with the documentation in a French study about an AmpC β-lactamase-hyperproducing *Enterobacter cloacae* in a university hospital (32). However, from the number of isolates recovered from the pediatric and neonatal floors, it might suggest that the non-adult wards, including the critical care ones, do not house significant pAmpCs. This will be interesting to investigate in the future. Although our data does not reflect a certain endemic situation or small separate epidemics, it does, however, show a relatively considerable presence of pAmpC. AmpC is considered a very rare entity mainly because, as previously mentioned, (1) many laboratories in Lebanon include AmpC under the category of ESBL, and (2) if it was isolated as separate, distinguishing plasmidic and chromosomal AmpC is very rarely done. As of late, no other study in Lebanon or the Arab World has looked into AmpC the way our study did, and despite the mentioned limitations, it can serve as a launching pad for further research work in the future.

## Conclusion

Nosocomial presence of AmpC β-lactamases is a major healthcare problem. Hospitals should implement firmer protocols that reach out to all of its units whether it is critical care units or any of its other wards without any discrepancies in the implementation. Our study further reinforces the claims to improve infection control among healthcare centers. Yet again, appropriate detection of any bacterial strain is vital to limit any of its development. A concordance between molecular and phenotypic strategies is in need for best detection of AmpC, chromosomal, and plasmid, especially with further needed improvement in the phenotypic detection domain which has recently shown high number of false positives. Routine screening for MDRs is a must since the mere thought of dissemination of any of these should be frightening to all medical centers, be it local or worldwide, as it does not only lead to possible outbreaks but also to possible therapeutic demise. We hope our study will be the first of many down the line concerning AmpC.

## Data Availability Statement

The original contributions presented in the study are included in the article/supplementary material, further inquiries can be directed to the corresponding author/s.

## Author Contributions

MF: data collection, data analysis, and manuscript writing. AM, OA, KH, DM, and EH: data collection. KM: data analyses and patients management. HS: data analysis and infection control color up. DS: molecular detection of AmpC data analysis and designed the molecular work related to the characterization of plasmid AmpC and performed the molecular experiments at Michigan Health Clinics Laboratories. ZD: project design, days analysis, and manuscript writing. All authors contributed to the article and approved the submitted version.

## Conflict of Interest

The authors declare that the research was conducted in the absence of any commercial or financial relationships that could be construed as a potential conflict of interest.

## Publisher's Note

All claims expressed in this article are solely those of the authors and do not necessarily represent those of their affiliated organizations, or those of the publisher, the editors and the reviewers. Any product that may be evaluated in this article, or claim that may be made by its manufacturer, is not guaranteed or endorsed by the publisher.
